# Diagnostic Accuracy of Hounsfield Unit Value and Hounsfield Unit to Hematocrit Ratio in Predicting Cerebral Venous Sinus Thrombosis: A Retrospective Case-Control Study

**DOI:** 10.7759/cureus.57567

**Published:** 2024-04-03

**Authors:** Chetana Ratnaparkhi, Avinash Dhok, Akhil Gupta, Amol Dube, Bheekam Kurmi, Ashwini Umredkar, Santha Kumar, Shilpa Pande, Samiksha Ghatol

**Affiliations:** 1 Department of Radiodiagnosis, All India Institute of Medical Sciences (AIIMS) Nagpur, Nagpur, IND; 2 Department of General Medicine, All India Institute of Medical Sciences (AIIMS) Nagpur, Nagpur, IND

**Keywords:** non-contrast computed tomography, cerebral venous sinus thrombosis, hounsfield unit/hematocrit ratio, hematocrit, hounsfield unit

## Abstract

Objective: Non-contrast computed tomography (CT) of the brain is a primary neuroimaging modality in emergency patients suspected of having cerebral venous sinus thrombosis (CVST). The objective of the study was to determine the diagnostic accuracy of Hounsfield unit (HU) values and the ratio of HU to hematocrit value (HU/Htc) in predicting CVST in suspected patients.

Material and methods: A retrospective, case-control study was done in a tertiary care institute which included 35 patients with CVST constituted as cases and 41 patients without CVST as controls on the basis of magnetic resonance venography (MRV). Non-contrast CT brain of all 76 subjects were assessed by two experienced radiologists independently. HU values of dural venous sinuses were calculated in both groups, and HU/Htc ratio was also determined. Statistical Package for Social Sciences (SPSS) version 25.0 (SPSS© for Windows, IBM© Corp.) was used for statistical analysis. Independent samples t-test was applied to compare the means of continuous variables. The diagnostic values were computed using the Calculator 1 tool on clinical research calculators tab on vassarstats.net. The predictive values of HU and HU/Htc ratio were estimated by the receiver operating characteristic (ROC) curve analysis.

Results: In CVST group, the mean Hounsfield Unit (HU) value was "75.9±3.9 (mean±SD)", while in control group, it was 57.78±4.65 (mean±SD), p < 0.001. The mean HU/Htc ratio was 1.98±0.42 (mean±SD) in the CVST group and 1.51±0.12 (mean±SD) in the control group (p < 0.001). Optimum cut-off HU value was calculated as 68, with 97% sensitivity and 100% specificity. For HU/Htc ratio, optimum cut-off was calculated as 1.69, yielding 71.4% sensitivity and 100% specificity on the basis of ROC curves. The difference was not statistically significant in hemoglobin and hematocrit (Htc) values between the cases and controls.

Conclusion: The quantitative measurements like HU value and HU/Htc ratio provide an easily obtainable metric in patients with suspected CVST on non-contrast CT brain, thus enhancing the role of non-contrast CT brain in diagnosing CVST.

## Introduction

Cerebral venous sinus thrombosis (CVST) is one of the important causes of stroke, particularly in the young population. It constitutes 10%-20% of strokes in young individuals and contributes 30% of stroke cases in India [1]. Even though there is advancement in imaging leading to improved rate of CVST detection, still delay in diagnosis occurs due to varied clinical presentation and gradual progression of the disease [2,3].

Contrast-enhanced magnetic resonance venography (MRV) is the preferred imaging for CVST detection. However, the majority of CVST patients present to the emergency department with acute neurological symptoms like headache, sensory or motor deficit, irritability, etc.; non-contrast computed tomography (CT) is done as a primary neuroimaging modality. However, sensitivity of non-contrast CT brain in detecting CVST in suspected patients is relatively less, and approximately 25%-30% of patients remain undiagnosed [4,5]. The commonest finding of CVST on non-contrast CT brain is hyperattenuation of dural venous sinuses (DVS) due to occlusion by the thrombus [6,7].

There are studies in the literature that emphasize on quantitative assessment of DVS by Hounsfield Unit (HU) values on CT brain in diagnosing CVST [8]. Furthermore, the attenuation value resulting from hemoglobin (Hb) changes in direct proportion to Hb and hematocrit (Htc) levels [8,9]. Considering this association, HU obtained from CT brain needs to be compared and interpreted in correlation with the patient’s Hb and Htc values. The current study aims to determine the diagnostic accuracy of HU values obtained on non-contrast CT brain scans and the ratio of HU/Htc in CVST patients in correlation with controls.

## Materials and methods

The current study is a retrospective, case-control study conducted in the Department of Radiodiagnosis of a tertiary healthcare center in central India. The approval was granted by the Institutional Review Board (IRB) and Institutional Ethics Committee (IEC) of All India Institute of Medical Sciences, Nagpur, with approval number, IEC/Pharmac/2024/759, dated February 8, 2024. The study duration was 18 months, from June 2022 to December 2023. Study population included patients presenting to the emergency department with headache and other neurological symptoms and diagnosed with CVST and without CVST on MRV or CT venography (CTV).

Inclusion criteria for cases were patients above 18 years of age presenting with acute neurological symptoms, including headache, sensory and motor deficit, irritability, vomiting to the emergency department, and diagnosed with CVST on MRV or CTV, and whose non-contrast CT scan brain was done as a primary neuroimaging test. Patients below 18 years of age were excluded as cases from the study. Inclusion criteria for controls were patients above 18 years of age, presenting in the emergency department with headache and whose non-contrast CT scan brain showed no CVST or brain parenchymal abnormality which were confirmed on CTV and MRV. Patients below 18 years of age were excluded as controls from the study. The study parameters, HU, Hb, Htc, and HU/Htc ratio, were evaluated in cases and controls.

All the CT brain scans were done on Siemens 256 slice dual source, dural energy CT scanner (Somatom Drive with Fluoroscopy) using CT brain protocol. The protocol was 344 mAs, 120 kVp, 5x1 mm slice thickness, 200 field of view (FOV), and pitch=0.45.

For CTV, intravenous low-osmolar iodinated contrast media containing 350 mg Iodine/ml was used in a dose of 1 ml/kg body weight with an injection rate of 4 ml/sec, followed by saline chase. All images were interpreted on Syngo.via software. Magnetic resonance imaging (MRI) was done on 3 Tesla MRI Siemens Magnetom Skyra. MRI brain and MRV were done in suspected CVST patients.

MRI and MRV sequences and their parameters were as follows: fluid-attenuated inversion recovery (time to repeat (TR)=8640, time to echo (TE)=81, inversion time (TI)=2457), T2 (TR=5500, TE=100), T1 (TR=2000, TE=9), diffusion-weighted imaging (TR=3600, TE=74), and susceptibility-weighted imaging (TR=27, TE=20). Dynamic contrast venography was done after injecting Gadolinium as contrast media intravenously in a dose of 0.1 mmol/kg at the rate of 4 ml/sec.

The CT findings for cases and controls were interpreted separately and randomly by two radiologists trained in emergency radiology reporting, and they were blinded for results. HU values of sigmoid sinuses, bilateral transverse sinuses, straight sinus, inferior sagittal sinus, and superior sagittal sinus were recorded by the radiologists without knowing the patient’s demographics and clinical history. In cases having additional dural sinus involvement other than the above-mentioned sinuses, the findings were mentioned separately. In cases showing irreconcilable results, mutual assessment was done and consensus about HU value was obtained. HU values were recorded with the help of region of interest (ROI) method. In the focal area of thrombosis, ROI was taken in comparison with MRV or CTV findings. In cases with multiple sinus involvement, the mean of the highest HU value was considered for analysis. In control group, the mean of the highest HU value of sinuses was considered for statistical analysis.

In CVST patients, intraparenchymal abnormalities, such as intraparenchymal hemorrhage, hemorrhagic or non-hemorrhagic infarct, and subarachnoid hemorrhage, were also included. Hb and Htc values were taken from digital records of the hospital information system of the institute. HU/Htc ratio is calculated by dividing HU values by Htc of patients in both cases and controls. Clinical details of cases were also documented.

Statistical methods

Statistical Package for Social Sciences (SPSS) version 25.0 (SPSS© for Windows, IBM© Corp.) was used for statistical analysis. For the normal distribution of continuous variables, a standard statistical test was employed. Continuous variables were presented as mean, standard deviation, and 95% confidence interval (CI). Categorical variables were mentioned in frequencies and rates. Independent samples t-test was applied to compare the means of continuous variables. The diagnostic values were computed using the Calculator 1 tool on clinical research calculators tab on vassarstats.net. The predictive values of HU and HU/Htc ratio were estimated by receiver operating characteristic (ROC) curve analysis. Area under curve (AUC) was also calculated. The correlation between HU and Htc values was evaluated by linear regression analysis. The above statistical methods were applied to ensure a comprehensive data evaluation and to draw meaningful conclusions from the analyses conducted.

## Results

In the current study, 76 patients were included. Out of 76 patients, 35 were in CVST group and 41 were in control group. In cases group, out of 35 CVST patients, males were 25 (71.5%) and females were 10 (28.5%). In control group, out of 41 patients, males were 31 (75.6%) and females were 10 (24.4%). The mean age in CVST was 39 years and in controls was 34.5 years.

Patients in CVST group presented with various neurological complaints. The commonest complaint was headache (71.4%) followed by altered sensorium (34.2%), motor deficit (28.5%), seizures (28.5%), and vomiting (28.5%). Other complaints were sensory deficit, blurring of vision, and irritability (Table 1).

**Table 1:** Neurological signs and symptoms in patients with cerebral venous sinus thrombosis

Clinical Signs and Symptoms	Number of Patients, n (%)
Headache	25 (71.4%)
Altered sensorium	12 (34.2%)
Motor deficit	10 (28.5%)
Seizures	10 (28.5%)
Vomiting	10 (28.5%)
Blurring of vision	6 (17.14%)
Sensory deficit	4 (11.4%)
Irritability	2 (5.7%)

Non-contrast CT scans of CVST patients showed various brain parenchymal findings. The commonest finding was hemorrhagic venous infarction which was noted in 14 patients (40%). Ten patients (28.5%) showed no abnormal findings on non-contrast CT brain (Table 2).

**Table 2:** Brain parenchymal findings in cerebral venous sinus thrombosis patients on non-contrast computed tomography

Non-contrast Computed Tomography Findings	Number of Patients, n (%)
Hemorrhagic venous infarction	14 (40%)
Intraparenchymal hemorrhage	5 (14.2%)
Subarachnoid hemorrhage	5 (14.2%)
Subdural hemorrhage	2 (5.7%)
Non-hemorrhagic infarction	4 (11.4%)
No abnormality	10 (28.5%)

MRV and CTV of cases showed superior sagittal sinus as commonly involved sinus in thrombosis followed by transverse and sigmoid sinus. Three patients showed thrombosis of cortical veins at the vertex, and eight patients showed thrombosis of the internal jugular vein as an additional finding (Table 3).

**Table 3:** Thrombosis site of various cerebral venous sinuses in patients with cerebral venous sinus thrombosis

Cerebral Venous Sinus Involved	No of Patients, n (%)
Superior sagittal sinus	15 (42.8%)
Transverse sinus	2 (5.7%)
Superior sagittal sinus + transverse sinus + sigmoid sinus	8 (22.85%)
Transverse sinus + sigmoid sinus	5 (14.2%)
Internal cerebral veins thrombosis + vein of Galen thrombosis	1 (2.8%)
Internal cerebral veins + vein of Galen + straight sinus	1 (2.8%)
Superior sagittal sinus+ straight sinus + transverse sinus + sigmoid sinus	1 (2.8%)
Internal cerebral veins + vein of Galen+ transverse sinus + sigmoid sinus	1 (2.8%)
Straight sinus + transverse sinus + sigmoid sinus	1 (2.8%)

**Figure 1:**
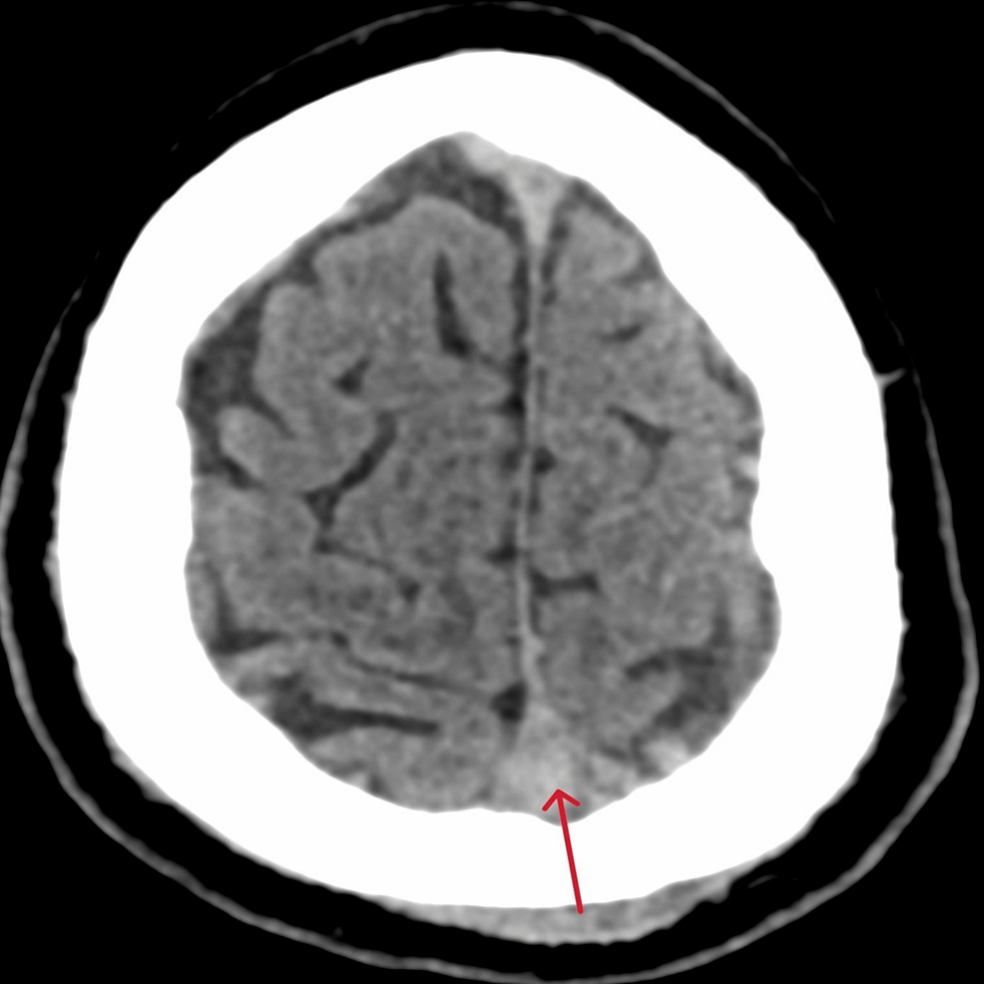
Hounsfield Unit value of non-contrast computed tomography of brain in cerebral venous sinus thrombosis Arrow shows hyperdense superior sagittal sinus with Hounsfield Unit value of 71.3.

**Figure 2:**
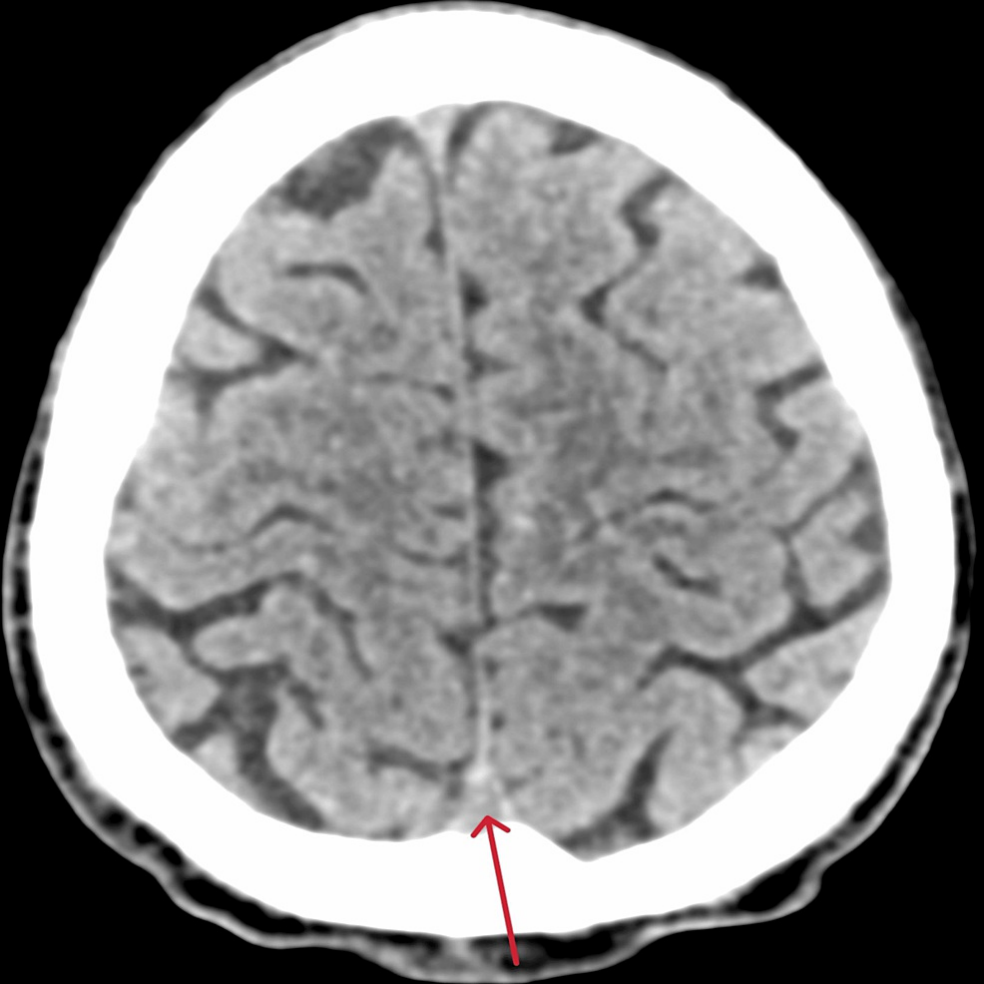
Hounsfield Unit value of non-contrast computed tomography of brain of control patient Arrow showing Hounsfield Unit value of 53.5 in superior sagittal sinus in control patient without thrombosis.

There is a significant difference in HU values of cases and controls (Figures 1, 2).

No statistically significant difference was noted between Hb and Htc values of controls. HU value and HU/Htc ratio of patients with CVST were comparatively higher. Significant correlation was noted in HU values and HU/Htc ratio of CVST cases and controls (p< 0.001) (Table 4).

**Table 4:** Hemoglobin (Hb), hematocrit (Htc), Hounsfield Unit (HU) values, and HU/Htc ratio in cerebral venous sinus thrombosis (CVST) patients and control groups p <0.001 statistically significant.

Parameter	CVST Patients (Cases), Mean±SD	Controls, Mean±SD	p-Value
Hb	12.9±2.0	12.6±2.2	0.53 (not significant)
Htc	39.3±6.0%	38.7±6.3%	0.66 (not significant)
HU	75.9±3.9	57.78±4.65	<0.001 (significant)
HU/Htc	1.98±0.42	1.51±0.12	<0.001 (significant)

**Figure 3:**
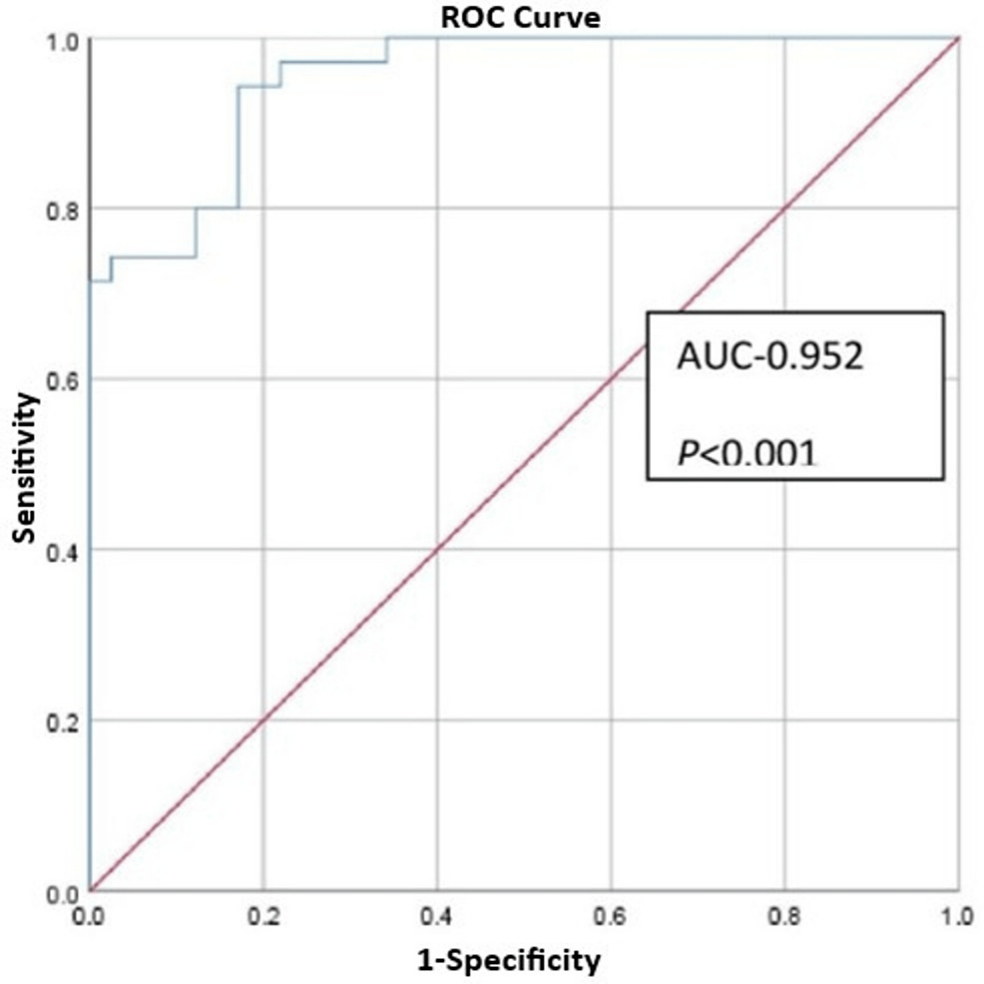
Receiver operating characteristic (ROC) curve showing area under curve (AUC) of Hounsfield Unit/hematocrit (HU/Htc) ratio

On linear regression analysis, a positive correlation was seen between Htc and HU values of controls (r=0.908, p<0.002), but no correlation was seen in CVST group (r=0.834, p<0.2). Analysis of ROC curve of HU/Htc ratio showed AUC of 0.952 (95% CI, 0.91-0.99; p <0.001) and optimum cut-off value of 1.69 for HU/Htc ratio showing sensitivity of 71.4% and specificity of 100% (Figure 3).

**Figure 4:**
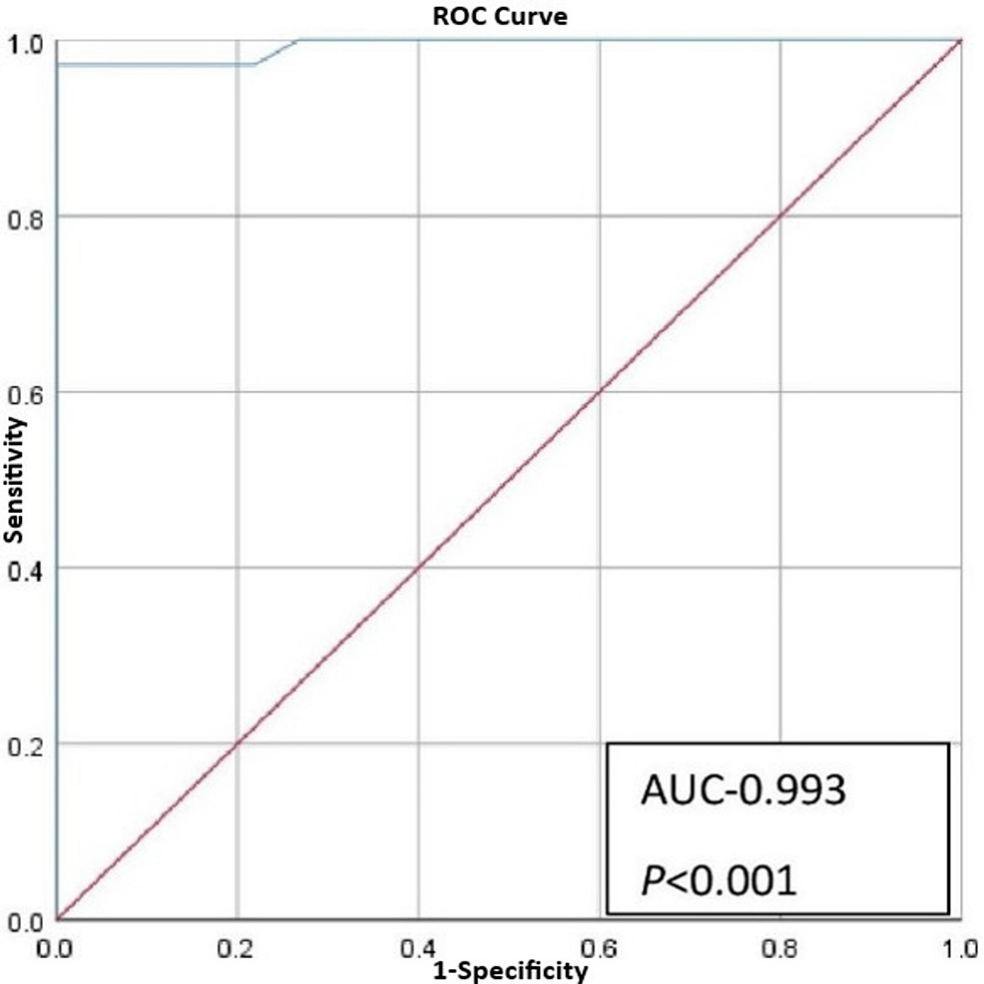
Receiver operating characteristic (ROC) curve showing area under curve (AUC) of Hounsfield Unit (HU)

Analysis of ROC curve of HU showed AUC of 0.993 (95% CI, 0.979-0.100; p<0.001) and optimum cut-off value of 68 showing sensitivity of 97% and specificity of 100% (Figure 4, Table 5).

**Table 5:** Diagnostic value analysis of optimum cut-off of Hounsfield Unit (HU)and Hounsfield Unit to hematocrit ratio (HU/Htc ratio) in cerebral venous sinus thrombosis (CVST) CI: confidence interval, LR: likelihood ratio, PPV: positive predictive value, NPV: negative predictive value.

	Sensitivity % (95% CI)	Specificity % (95% CI)	LR+ (95% CI)	LR- (95% CI)	PPV % (95% CI)	NPV % (95% CI)
HU >68	97 (83-99)	100 (89-100)	-	0.028 (0.004-0.197)	100 (87-100)	97 (85-99)
HU/Htc >1.69	71.4 (53-84)	100 (89-100)	-	0.285 (0.169-0.482)	100 (83-100)	80 (66-89)

## Discussion

Non-contrast CT brain is the first-line imaging modality in emergency situation for majority of the neurological symptoms. Compared to MRI, CT brain is cost-effective and less time-consuming. In emergency situations, where patients are mostly unstable, we need to rely on non-contrast CT findings for further management. In CVST, the commonest non-contrast CT brain finding is hyperdense dural venous sinuses which is a qualitative assessment leading to a false positive diagnosis; hence, patients will be subjected to further imaging which will lead to unnecessary delay in treatment. The quantitative method that is available in these patients is a measurement of HU value which helps to eliminate subject bias in qualitative assessment. In our study, we have calculated HU values of patients with ROI method and obtained HU/Htc ratio which is quick and easy to calculate in patients presenting to the emergency department, thus avoiding further unnecessary imaging and delay in effective management.

The most common symptom in CVST is headache which was present in 25 (71.4%) CVST patients. Hence, the controls were selected as patients with headache attending to emergency department. In the current study, the mean age in CVST cases was 39 years and in controls was 34.5 years, which was comparable between the cases and controls.

Traditionally, hyperdense dural venous sinuses are the marker of CVST. However, this qualitative assessment leads to a false negative diagnosis and is not present in all CVST patients [3,10]. Earlier studies by Roland et al., Zaheer et al., and Linn et al. reported sensitivity ranging from 63% to 73% in the detection of hyperdense sinuses in CVST patients on non-contrast CT brain studies [2,6,11]. Studies by Zaheer et al. and Digge et al. observed that quantitative assessment of dural sinus attenuation will likely enhance the detection rate of CVST in non-contrast CT brain [6,12].

As per the study by Buyck et al., attenuation of dural venous sinuses above 62 HU had sensitivity of 95% and specificity of 95% for the diagnosis of CVST [8]. As per the study by Besachio et al., attenuation of dural venous sinuses above 65 HU showed sensitivity and specificity of 84% and 96%, respectively, in diagnosing CVST [13]. Studies by Black et al. and Digge et al. showed a cut-off HU value for the diagnosis of CVST as 70 [9,12]. In a study published by Tayyebi et al., the predictive value of HU of 61 showed 82% sensitivity and specificity of 100% in diagnosing CVST [14].

In the current study, the HU value in CVST patients was found significantly higher statistically than in controls (p<0.001). The predictive value of dural venous sinus attenuation for CVST was 68 HU which showed sensitivity of 97% and 100% specificity. Our results are in concordance with the study by Tayyebi et al. [14].

The commonest brain parenchymal abnormality was hemorrhagic venous infarction which was seen in 40% of CVST patients. This finding warrants careful evaluation of dural venous sinuses. We observed that the commonest sinus involved in CVST was the superior sagittal sinus, in isolation or combination with other sinuses.

Apart from HU value calculation, another quantitative assessment of dural venous sinuses is HU/Htc ratio. There are limited studies on HU/Htc ratio in the available literature. In a study conducted by Black et al., on HU/Htc ratio of CVST patients and controls, the mean HU/Htc ratio in CVST group was 2.2 and in control was 1.44 [9]. They included eight patients in each group. In a study by Besachio et al., HU/Htc of 1.7 showed sensitivity of 56% and specificity of 96% [13]. In a study conducted by Buyck et al. [8], there were 20 patients each in CVST and controls and HU/Htc ratio was 1.9 in CVST group and 1.3 in controls. The difference they observed was statistically significant [8].

In the present study, HU/Htc ratio was higher in CVST group which is consistent with previous studies. The predictive value of HU/Htc ratio for diagnosing CVST was >1.69 showing 71.4% sensitivity and 100% specificity and p<0.001. This indicates that with a higher HU/Htc ratio, the probability of having CVST is more and these patients should be evaluated with further imaging like CTV or MRV.

However, a lower HU/Htc ratio will not exclude CVST. The dural venous sinus attenuation can be affected by various factors which include the patient’s age, hydration status, duration of sinus thrombosis, size of thrombus, and Htc values. In a study done by Akhavan et al., a negative correlation was observed between blood urea nitrogen/creatinine ratio and HU value suggesting that the hydration of a patient is an important factor that determines the HU values of dural venous sinuses [15].

There is a positive correlation between the patient’s age and HU value. In contrast, Htc value demonstrated a negative correlation [16]. Thus, age needs to be considered while assessing young patients with CVST. Also, hyperattenuation of thrombus decreases with increasing duration of thrombus which should be considered while evaluating CVST patients with HU and HU/Htc ratio. So HU values and HU/Htc ratio may be misleading in chronic dural sinus thrombosis [17].

A positive correlation between HU and Htc of control group was found in the present study, whereas no correlation was observed in CVST group which is consistent with results of previous studies done by Buyck et al., Akhavan et al., and Canakci et al. [8,15,17]. In patients having increased Htc, dural venous sinuses may appear hyperattenuated leading to false positive results [17]. Adding HU/Htc ratio values can contribute to a more precise assessment, reducing the likelihood of false positives and enhancing the overall reliability of the diagnostic methods.

In the present study, 10 patients in CVST group showed no pathological findings on non-contrast CT. However, all these 10 patients showed HU value >68 and HU/Htc ratio >1.69. So the correlation between HU values and HU/Htc ratio proved problem-solving in these patients while reaching to accurate diagnosis.

Study limitations

Being a retrospective study, there could be selection bias. However, the selection method was the same as in previous studies [2,8,17]. The confounders affecting dural sinus attenuation are not considered as this is a retrospective study. The sample size was limited; hence, multi-center, prospective studies with more sample size on this topic will provide greater validity.

## Conclusions

MRV and CTV are imaging techniques for precise diagnosis of CVST. The quantitative measurements such as HU and HU/Htc ratio provide an easily obtainable metric in patients with suspected CVST on non-contrast CT brain, thus enhancing the role of non-contrast CT brain in diagnosing CVST. In cases where HU is greater than 68 and the HU/Htc ratio is more than 1.69, further imaging with CTV/MRV is recommended.

These quantitative measurements are valuable for clinicians in the diagnosis of CVST. The ability to perform an accurate evaluation in a short timeframe based on these measurements proves beneficial. These quantitative measurements can contribute to the reduction in unnecessary further imaging examinations, streamlining the diagnostic process and improving the overall efficiency of patient care. It will be helpful in resource-constrained areas of developing countries where accessibility to advanced imaging like MRI is limited and quick and cost-effective results can be generated with the help of HU values and HU/Htc ratio in patients with CVST.
